# Early pharmacological venous thromboembolism prophylaxis reduces lower limb deep vein thrombosis in surgical patients with severe hypertensive intracerebral hemorrhage: a retrospective cohort study

**DOI:** 10.3389/fneur.2026.1800147

**Published:** 2026-04-10

**Authors:** ZhongXin Yang, ZongXi Li, TianQuan Zhao, Kai Yu, Lie Zhang, Xun Xia

**Affiliations:** Department of Neurosurgery, Clinical Medical College, The First Affiliated Hospital of Chengdu Medical College, Chengdu, China

**Keywords:** deep vein thrombosis, early postoperative period, low-molecular-weight heparin, severe hypertensive intracerebral hemorrhage, venous thromboembolism prophylaxis

## Abstract

**Objective:**

To investigate the efficacy and safety of early pharmacological prophylaxis for venous thromboembolism (VTE) on the incidence of lower extremity deep vein thrombosis (DVT) in patients undergoing surgery for severe hypertensive intracerebral hemorrhage (HICH).

**Methods:**

In this retrospective cohort study, 123 surgical patients with severe HICH admitted between September 2021 and February 2025 were included. Based on the initiation timing of VTE pharmacological prophylaxis (low-molecular-weight heparin calcium, LMWH), patients were categorized into an early group (prophylaxis initiated within <48 h after admission) and a late group (prophylaxis initiated at ≥48 h after admission). To compare the incidence of lower extremity deep vein thrombosis (DVT), intracranial rebleeding, and gastrointestinal bleeding within one week after prophylaxis initiation, as well as the 60-day mortality rate and 60-day modified Rankin Scale (mRS) score, between the two groups.

**Results:**

The VTE prophylaxis initiation times were (29.06 ± 4.44) hours and (70.27 ± 10.12) hours in the early and late groups, respectively. The incidence of lower extremity DVT was significantly lower in the early group compared to the late group (17.5% vs. 38.3%, *p* < 0.05). No statistically significant differences were observed between the two groups in the incidence of intracranial rebleeding (9.5% vs. 5.0%), gastrointestinal bleeding (23.8% vs. 21.7%), 60-day mortality (14.3% vs. 11.7%), or 60-day modified Rankin Scale (mRS) score [4 (IQR 2–5) vs. 4 (IQR 2–5)] (all *p* > 0.05).

**Conclusion:**

For surgical patients with severe HICH, early postoperative (<48 h) pharmacological VTE prophylaxis was associated with a significantly reduced risk of lower extremity DVT, and no significant increases in intracranial rebleeding, gastrointestinal bleeding, or mortality were observed. However, given the limited sample size and number of events, these safety findings should be interpreted with caution.

## 1 Introduction

Hypertensive intracerebral hemorrhage (HICH) is a severe form of stroke. Despite surgical intervention effectively lowering intracranial pressure and reducing mortality, postoperative complications remain frequent, contributing to high rates of disability and mortality (1, 2). These patients often require prolonged bed rest due to impaired consciousness and limb paralysis, compounded by surgical trauma and systemic stress, leading to a hypercoagulable state and a significantly elevated risk of venous thromboembolism (VTE) (3). Lower extremity deep vein thrombosis (DVT) is a common complication, which not only may cause limb dysfunction, but more critically, can lead to fatal pulmonary embolism upon thrombus dislodgement, posing a severe threat to patient survival. Therefore, effective VTE prophylaxis is paramount.

Preventive measures for VTE include mechanical and pharmacological prophylaxis, both of which are effective in reducing the incidence of DVT (4–6). Despite guidelines recommending consideration of pharmacological prophylaxis for patients with ICH (7), concerns regarding intracranial rebleeding often lead to delayed or avoided administration of anticoagulants in clinical practice, particularly in severe HICH patients undergoing neurosurgery who are at even higher risk of rebleeding (8). Consequently, there is a lack of high-level evidence guiding the optimal timing for initiating VTE pharmacological prophylaxis in this specific population, resulting in significant variation in clinical practice. Therefore, this retrospective cohort analysis aims to investigate the impact and safety of early postoperative initiation of VTE pharmacological prophylaxis on the incidence of lower extremity DVT in surgical patients with severe HICH, thereby providing evidence to inform clinical decision-making.

## 2 Materials and methods

### 2.1 Study design and participants

This single-center retrospective cohort study consecutively enrolled surgical patients with severe HICH admitted to the Department of Neurosurgery, The First Affiliated Hospital of Chengdu Medical College, between September 2021 and February 2025.

*Inclusion criteria*: (1) Diagnosis of HICH; (2) Supratentorial hemorrhage; (3) Glasgow Coma Scale (GCS) score ≤ 8 on admission; (4) Undergone craniotomy for hematoma evacuation (with or without decompressive craniectomy).

*Exclusion criteria*: (1) Hospital admission >24 h after symptom onset; (2) Presence of respiratory or circulatory failure on admission; (3) History of hematological diseases; (4) Preoperative coagulation abnormalities or long-term use of oral anticoagulants/antiplatelet agents; (5) Hemorrhage secondary to aneurysm, arteriovenous malformation, or tumor; (6) Intraoperative death or withdrawal of life-sustaining treatment by family postoperatively; (7) Pre-existing lower extremity DVT confirmed by ultrasound before anticoagulant prophylaxis initiation; (8) History of active peptic ulcer disease.

Patients were divided into two groups based on the timing of VTE pharmacological prophylaxis initiation: the Early Group (initiation <48 h after admission) and the Late Group (initiation ≥48 h after admission). This study was conducted in accordance with the Declaration of Helsinki (2013 revision) and was approved by the Ethics Committee of The First Affiliated Hospital of Chengdu Medical College (Approval No. 2025CYFYIRB-BA-162). Informed consent was waived by the ethics committee due to the retrospective nature of the study.

### 2.2 Treatment protocol

All patients underwent preoperative cerebrovascular imaging (CTA or DSA) to rule out secondary causes of hemorrhage. Hematoma volume was calculated using the ABC/2 formula. Patients underwent microscopic hematoma evacuation with or without decompressive craniectomy (DC) based on clinical status. Postoperative management included blood pressure control, intracranial pressure management, glycemic control, temperature management, and infection prophylaxis. All patients undergoing surgery routinely underwent D-dimer testing on the first postoperative day. When the D-dimer level exceeded 243 ng/mL (the upper limit of the normal reference range at our institution), lower extremity compression ultrasonography was performed. If the ultrasound result was negative, pharmacological VTE prophylaxis was initiated. If DVT was confirmed, a vascular surgery consultation was obtained for treatment planning, and the patient was not included in the prophylactic medication analysis. All patients also routinely underwent cranial CT after surgery, and low-molecular-weight heparin was initiated only after CT confirmed hematoma stability (i.e., no active bleeding or significant hematoma expansion). This safety checkpoint was uniformly applied to all patients.

#### 2.2.1 VTE pharmacological prophylaxis regimen

Low-molecular-weight heparin calcium (LMWH) at a dose of 5,000 IU was administered subcutaneously once daily. During prophylaxis, ultrasound was performed promptly if clinical suspicion of DVT arose. Prophylaxis was discontinued upon diagnosis of DVT. LMWH was also immediately discontinued if intracranial rebleeding or clinically significant gastrointestinal bleeding occurred.

### 2.3 Outcome measures

*Primary outcome*: The incidence of new-onset lower extremity DVT assessed by venous duplex ultrasound on day 7 after prophylaxis initiation, including thrombus distribution (proximal veins: common femoral, deep femoral, popliteal; distal veins: anterior tibial, posterior tibial, peroneal, soleal veins).

*Secondary outcomes*: The incidences of intracranial rebleeding and gastrointestinal bleeding during the VTE prophylaxis period.60-day mortality and 60-day modified Rankin Scale (mRS).

*Intracranial rebleeding*: Defined as a new hemorrhage or an increase in the original hematoma volume by >33% compared to the first postoperative CT scan, as independently assessed by a neurosurgeon and a radiologist.

*Gastrointestinal bleeding* (9): Defined according to prior criteria as the presence of coffee-ground or bloody gastric aspirate, melena, or a positive fecal occult blood test.

### 2.4 Data collection

Collected data included baseline characteristics (age, gender, body mass index), clinical parameters [platelet count (PLT), prothrombin time (PT), activated partial thromboplastin time (APTT), D-dimer, comorbidities, admission GCS score], hemorrhage characteristics (location, volume), surgical procedure, presence of femoral venous catheterization, timing of VTE prophylaxis initiation, and all outcome measures.

### 2.5 Statistical analysis

Statistical analysis was performed using SPSS software (version 26.0). Normally distributed continuous data are presented as mean ± standard deviation (SD) and compared using the independent samples t-test. Categorical data are presented as number (percentage) and compared using the Chi-square test or Fisher’s exact test, as appropriate. Since the parallel lines test for the ordinal logistic regression yielded a *p*-value <0.05, indicating that the proportional odds assumption was violated, the Mann–Whitney U test was used for intergroup comparison of mRS. Continuous data are expressed as median (interquartile range, IQR). Binary logistic regression was used to assess the effect of early LMWH initiation (<48 h vs. ≥48 h) on lower extremity DVT, as well as the effects of early LMWH initiation (<48 h vs. ≥48 h) and lower extremity DVT on mortality, with adjusted odds ratios (OR) and their 95% confidence intervals (CI) calculated. Based on clinical importance and univariable analysis results, age, admission GCS score, hemorrhage volume, and femoral venous catheterization were included in the model as covariates for adjustment. Model goodness-of-fit was evaluated using the Hosmer–Lemeshow test. For intracranial rebleeding and gastrointestinal bleeding, due to the limited number of events and thus limited statistical power, only univariable analyses (χ^2^ test or Fisher’s exact test) were performed, and the results are presented as exploratory analyses without multivariable adjustment. All tests were two-tailed, and a *p*-value < 0.05 was considered statistically significant.

## 3 Results

### 3.1 Baseline characteristics

A total of 123 patients were included: 63 in the Early Group and 60 in the Late Group. The VTE prophylaxis initiation times were (29.06 ± 4.44) hours and (70.27 ± 10.12) hours for the Early and Late Groups, respectively (*p* < 0.001). Time interval from end of surgery to initiation of VTE prophylaxis were (16.62 ± 4.14) hours and (55.87 ± 9.67) hours for the Early and Late Groups, respectively (*p* < 0.001). No significant differences were observed between the two groups regarding age, gender, BMI, baseline coagulation parameters, comorbidities (hypertension, diabetes), hemorrhage location and volume, surgical procedure, preoperative GCS score, rate of femoral venous catheterization, or postoperative D-dimer levels (all *p* > 0.05), indicating balanced baseline characteristics (Table 1).

**Table 1:** Comparison of baseline characteristics between the early and late groups.

Variables	Early group *n* = 63	Late group *n* = 60	t/χ^2^	*p* value
Age (y)	52.86 ± 15.00	54.58 ± 12.05	−0.701	0.484
Sex (male)	27 (42.9)	31 (51.7)	0.957	0.328
BMI (kg/m^2^)	25.45 ± 1.72	25.22 ± 1.92	0.701	0.485
PLT (10^9^/L)	172.52 ± 52.55	163.87 ± 52.40	0.915	0.362
PT (s)	14.03 ± 1.06	13.79 ± 1.14	1.179	0.241
APTT (s)	30.24 ± 2.96	29.25 ± 2.76	1.916	0.058
Hypertension	52 (82.5)	49 (81.7)	0.016	0.900
Type 2 Diabetes	37 (58.7)	34 (56.7)	0.054	0.817
Hemorrhage location				
Basal ganglia region	43 (68.3)	35 (58.3)	1.304	0.254
Lobar	20 (31.7)	25 (41.7)		
Hemorrhage volume (mL)	63.37 ± 11.88	66.60 ± 11.93	−1.507	0.134
Surgical procedure				
Hematoma evacuation	29 (46.0)	30 (50.0)	0.194	0.660
Hematoma evacuation +DC	34 (54.0)	30 (50.0)		
D-dimer (ng/mL)	723.03 ± 320.98	769.85 ± 276.70	−0.865	0.389
VTE Prophylaxis Initiation Time (h)	29.06 ± 4.44	70.27 ± 10.12	−29.485	0.000
Time interval from end of surgery to initiation of VTE prophylaxis (h)	16.62 ± 4.14	55.87 ± 9.67	−29.51	0.000
GCS (points)	5.49 ± 1.37	5.37 ± 1.54	0.478	0.633
Femoral venous catheterization	54 (85.7)	50 (83.3)	0.133	0.715

Reference range: BMI: 18.5–23.9 kg/m^2^; PLT: 125–300 × 10^9^/L; PT: 9.4–12.5 s; APTT: 25.1–36.5 s; D-dimer: 0–243 ng/mL.

Multivariable logistic regression analysis showed that after adjusting for age, hemorrhage volume, GCS, and femoral venous catheterization, early initiation of LMWH (<48 h) remained significantly associated with a reduced risk of lower extremity DVT (OR = 0.278, 95% CI: 0.111–0.694, *p* = 0.006). GCS was marginally negatively associated with DVT risk (OR = 0.747, 95% CI: 0.552–1.010, *p* = 0.058), and age showed a marginal association (OR = 0.969, 95% CI: 0.937–1.001, p = 0.058). Hemorrhage volume and femoral venous catheterization were not significantly correlated with DVT (*p* > 0.05) (Table 2).

**Table 2:** Multivariable logistic regression analysis for lower extremity DVT, adjusted for age, GCS, hemorrhage volume, and femoral venous catheterization.

Variables	*β*	SE	Wald χ^2^	*p* value	OR	95%CI
Age	−0.032	0.017	3.580	0.058	0.969	0.937 ~ 1.001
Hemorrhage volume	−0.017	0.019	0.855	0.355	0.983	0.947 ~ 1.020
GCS	−0.292	0.154	3.597	0.058	0.747	0.552 ~ 1.010
Femoral venous catheterization (yes vs. no)	0.254	0.573	0.196	0.658	1.289	0.419 ~ 3.962
Groups (early vs. late)	−1.280	0.467	7.514	0.006	0.278	1.111 ~ 0.694

Group (early vs. late) was coded as early = 1, late = 0; femoral venous catheterization (yes vs. no) was coded as yes = 1, no = 0. Hosmer–Lemeshow test, *p* = 0.627.

### 3.2 Outcome comparison

#### 3.2.1 Lower extremity DVT

Among the 123 patients, 34 developed lower extremity DVT (overall incidence 27.6%), comprising 11 proximal and 23 distal DVTs. Multivariable logistic regression analysis showed that after adjusting for age, GCS score, hemorrhage volume, and femoral venous catheterization, early LMWH initiation (<48 h) was associated with a significantly lower risk of lower extremity DVT (adjusted OR = 0.28, 95% CI 1.11–0.69, *p* = 0.006). Univariable analysis also revealed a significantly lower incidence of DVT in the early group compared with the late group (17.5% vs. 38.3%, unadjusted OR = 2.94, 95% CI 1.28–6.76, *p* = 0.010). Among patients who developed DVT, the distribution of proximal and distal vein thrombosis did not differ significantly between the two groups (*p* = 0.730) (Table 3).

**Table 3:** Comparison of outcomes between the early and late groups (*n* = 123).

Outcome	Early group *n* = 63	Late group *n* = 60	Unadjusted OR (95% CI)	Adjusted OR (95% CI)^*^	*p* value (unadjusted)	*p* value (adjusted)
Lower Limb DVT	11 (17.5)	23 (38.3)	2.94 (1.28 ~ 6.76)	0.28 (1.11 ~ 0.69)	0.010	0.006
Proximal veins	4 (36.4)	7 (30.4)		−+	0.730	−+
Distal veins	7 (63.6)	16 (69.6)		−+		−+
Intracranial rebleeding	6 (9.5)	3 (5.0)	2.00 (0.48 ~ 8.39)	−+	0.336	−+
Gastrointestinal bleeding	15 (23.8)	13 (21.7)	0.89 (0.38 ~ 2.06)	−+	0.777	−+
60-day mortality	9 (14.3)	7 (11.7)	0.79 (0.28 ~ 2.28)	0.58 (0.17 ~ 2.05)	0.666	0.397
60-day mRS (M, IQR)	4 (2 ~ 5)	4 (2 ~ 5)	−+	−+	0.965	−+

^*^Lower Limb DVT: Adjusted for age, GCS, hemorrhage volume, and femoral venous catheterization.60-day mortality: Adjusted for age, GCS, lower limb DVT, and VTE prophylaxis initiation time. Unadjusted OR not applicable for mRS; group comparison by Mann–Whitney U test (*Z* = −0.044, *p* = 0.965). −+: Multivariable analysis not performed due to limited events.

#### 3.2.2 Intracranial rebleeding and gastrointestinal bleeding

Due to the limited number of events, multivariable analysis was not performed for these safety outcomes, and only univariable analyses were conducted. There were no significant differences between the two groups in the incidence of intracranial rebleeding (9.5% vs. 5.0%, OR = 2.00, 95% CI 0.48–8.39, *p* = 0.336) or gastrointestinal bleeding (23.8% vs. 21.7%, OR = 0.89, 95% CI 0.38–2.06, *p* = 0.777) (Table 3).

#### 3.2.3 60-day mortality

Multivariable logistic regression analysis, adjusted for age, GCS, lower extremity DVT, and timing of VTE prophylaxis initiation, showed no significant difference in 60-day mortality between the early and late groups (adjusted OR = 0.58, 95% CI 0.17–2.05, *p* = 0.397). Univariable analysis also revealed no significant difference between the groups (14.3% vs. 11.7%, unadjusted OR = 0.79, 95% CI 0.28–2.28, *p* = 0.666) (Table 3).

#### 3.2.4 Functional outcome at 60 days (mRS)

The median 60-day mRS score was 4 (IQR 2–5) in both the early and late groups. The Mann–Whitney U test showed no significant difference between the groups (*Z* = −0.044, *p* = 0.965).

## 4 Discussion

This retrospective cohort study found that for surgical patients with severe HICH, early initiation (within 48 h) of VTE pharmacological prophylaxis based on LMWH significantly reduced the incidence of lower extremity DVT within one week postoperatively compared to delayed initiation (≥48 h) (17.5% vs. 38.3%), without a significant increase in the risks of intracranial rebleeding or gastrointestinal hemorrhage. This provides important evidence supporting the early and safe implementation of thromboprophylaxis in this high-risk population.

HICH is a devastating form of stroke, with a 30-day mortality rate ranging from 30 to 55% (10). Although advancements in surgical techniques and multimodal management have significantly reduced mortality and improved survival and outcomes—particularly in severe cases—the condition continues to impose a substantial economic and psychological burden on patients, families, and society due to its persistently high rates of fatality, disability, and severe functional impairment (11). Postoperative DVT is highly prevalent in severe HICH, with reported incidences of 20 to 40% in the literature (12, 13). Once dislodged, thrombi may migrate via the bloodstream to the lungs, potentially causing fatal pulmonary embolism. In this study, the overall DVT incidence was 27.6% (34/123), which aligns with the previously reported range.

Risk factors for postoperative DVT in patients with ICH include large hemorrhage volume, limb immobility, age >40 years, use of dehydrating agents, lower extremity central venous catheters, hypertension, diabetes, elevated BMI, hypercoagulability, and coagulation abnormalities following hemorrhage (14, 15)—factors nearly ubiquitous in patients with severe HICH undergoing surgery. Hypercoagulability represents a core pathophysiological mechanism for DVT formation, characterized by blood stasis. Animal models of VTE demonstrate that increased flow obstruction and reduced collateral circulation significantly elevate thrombus incidence and size (16). Furthermore, stasis-induced venous wall hypoxia may exacerbate VTE by promoting inflammation (17). Growing evidence also suggests that altered hemodynamics influence venous endothelial and smooth muscle cell phenotypes, thereby promoting thrombosis (18, 19). Postoperative severe HICH patients are typically hypercoagulable due to: (1) impaired consciousness and mobility, reducing lower limb venous return; (2) hemoconcentration from aggressive dehydration therapy for cerebral edema; and (3) endothelial injury and a systemic inflammatory response triggered by the hematoma, which activates coagulation, increases fibrinolysis, and elevates D-dimer levels (20). The markedly elevated postoperative D-dimer levels observed in both groups of our study confirm this early hypercoagulable state, providing a theoretical basis for early pharmacological intervention.

Although early mechanical prophylaxis can reduce the incidence of DVT in patients with ICH by approximately 50% (5, 6), a considerable number of patients still progress to DVT. For patients with severe HICH after surgery, the primary concern among clinicians regarding early initiation of pharmacological prophylaxis is the potential to induce or exacerbate bleeding. For instance, a cohort study of surgically treated traumatic brain injury patients found that pharmacological VTE prophylaxis within the first 3 days postoperatively reduced thromboembolic risk but increased the risk of reoperation (3). Therefore, the optimal timing for initiating VTE pharmacological prophylaxis after ICH remains a challenge in clinical decision-making.

Current guidelines, including the 2020 Chinese Stroke Association guidelines (21), the 2020 Canadian Stroke Best Practice Recommendations (22), and the 2022 American Heart Association/American Stroke Association guidelines (23), suggest that early use of low-dose unfractionated heparin and/or LMWH may be considered to reduce VTE risk in patients with ICH, provided that bleeding has ceased. However, these guidelines do not specify a concrete time point for “early” initiation. Furthermore, their recommendations are primarily based on evidence from non-surgical ICH patients, and the quality of evidence supporting some recommendations is limited. Given this clinical context and evidence gap, this study selected 48 h after admission as the cutoff point to divide patients into an early group and a late group. The rationale is as follows: ① Clinical observation suggests that hemorrhage in most severe HICH patients tends to stabilize by 48 h after admission, with a relatively lower risk of rebleeding. ② Such patients enter a hypercoagulable state early postoperatively. ③ The timing of the first postoperative head CT review varies among patients, and its findings are crucial for deciding whether to initiate pharmacological prophylaxis. ④ Few existing studies have used 48 h as a time node for investigation. In this study, the time of VTE prophylaxis initiation in the early and late groups was (29.06 ± 4.44) hours and (70.27 ± 10.12) hours, respectively, and the interval from the end of surgery to VTE prophylaxis initiation in the two groups was (16.62 ± 4.14) hours and (55.87 ± 9.67) hours, respectively, with significant differences. The DVT incidence was 17.5% in the early group, significantly lower than the 38.3% in the late group (*p* < 0.05). However, there was no significant difference in the distribution of thrombus locations between the two groups. Regarding safety, the intracranial rebleeding rates were 9.5 and 5.0% in the early and late groups, respectively, with no statistically significant difference. This aligns with the 9.2% rebleeding rate reported by Diao H et al. (15), and none of the rebleeding events required surgical re-intervention. The incidences of gastrointestinal bleeding were 23.8 and 21.7% in the early and late groups, respectively, slightly higher in the early group but without statistical significance (*p* > 0.05). These findings are consistent with recent studies on non-surgical intracerebral hemorrhage patients, which concluded that early initiation (within 24–48 h of admission) of prophylactic anticoagulation did not significantly increase the risk of hematoma expansion (24). In summary, the results of this study suggest that initiating prophylactic- LMWH within 48 h may be safe and effective, provided that surgical hematoma evacuation has been performed and the first postoperative CT confirms no active bleeding. Choosing 48 h as the time node balances the natural stabilization process of the hematoma in the early postoperative period for most patients with the practical feasibility of obtaining imaging evidence and making treatment decisions in clinical practice. In this study, the median interval from surgery to LMWH initiation was 16.6 h in the early group, substantially shorter than the 55.9 h in the late group. This early postoperative window aligns with the period when hypercoagulability is most pronounced following surgical stress, potentially explaining the observed reduction in DVT risk with early prophylaxis. Importantly, LMWH was initiated only after routine postoperative CT (performed within 24 h after surgery) confirmed hematoma stability, ensuring that pharmacological prophylaxis was not administered in the presence of active bleeding. This mandatory safety checkpoint likely contributed to the absence of a statistically significant increase in intracranial rebleeding in the early group. These detailed timing data enhance the clinical applicability of our findings by providing a clear temporal framework for early pharmacological VTE prevention in this high-risk population.

This study employed a retrospective design, and the timing of LMWH initiation was determined by clinicians based on patients’ postoperative status, initial cranial CT findings, and bleeding risk assessment. Therefore, confounding by indication may exist, meaning that patients who received early prophylaxis may have been clinically more stable or had a lower perceived bleeding risk, potentially contributing to a lower incidence of DVT. To address measurable confounders, a multivariable logistic regression model was used, adjusting for age, GCS, hemorrhage volume, and femoral venous catheterization, all of which are known to be associated with DVT. The adjusted results indicated that early LMWH initiation remained independently associated with a significantly reduced risk of DVT (OR = 0.28, 95% CI 0.111–0.694, *p* = 0.006), suggesting that the protective effect of early pharmacological intervention was not fully explained by the aforementioned confounders. Nevertheless, unmeasured confounders may still exist, such as precise assessment of hematoma stability on postoperative CT, intraoperative hemostatic efficacy, and individualized bleeding risk evaluation. These factors may concurrently influence clinicians’ decisions regarding the timing of LMWH initiation and patients’ risk of bleeding or thrombosis. Therefore, the potential for residual confounding inherent to the retrospective design should be carefully considered when interpreting the findings of this study.

Regarding the choice of pharmacological agents, there is currently no universally recommended regimen. This study employed a fixed-dose regimen of LMWH at 5000 IU administered subcutaneously once daily. Compared to unfractionated heparin or higher-dose LMWH regimens, this protocol may offer more stable pharmacokinetic properties and a lower bleeding risk in neurosurgical critical care patients (25, 26). Over the past decade, a major advancement in VTE management has been the introduction of direct oral anticoagulants (DOACs). Compared to previous heparin and vitamin K antagonist-based regimens, DOACs have demonstrated non-inferior efficacy with a superior safety profile (27–29), offering a favorable risk–benefit balance for extended-duration anticoagulation in VTE prevention (30, 31). However, despite their widespread use in VTE treatment, DOACs are less suitable for initiating prophylaxis in the early postoperative neurosurgical setting. This is due to their relatively long half-lives, unpredictable gastrointestinal absorption and hepatic/renal metabolism in critically ill patients, and the lack of immediate, universally available specific reversal agents in the context of acute intracranial hemorrhage. In contrast, subcutaneous LMWH presents distinct advantages in controllability during this vulnerable period. Its effects have a relatively rapid onset and offset, its dose–response relationship is more predictable, and it has a partial reversal agent available (protamine). These characteristics make LMWH a more manageable option for early prophylaxis following neurosurgery.

## 5 Limitations

Several limitations should be acknowledged in this study. First, this was a single-center retrospective study with inherent selection and information biases. Second, DVT screening was triggered by elevated D-dimer levels, which may have missed asymptomatic cases. Third, the sample size was modest, limiting statistical power for safety outcomes. Fourth, we lacked systematic data on pulmonary embolism—a rare but critical event—and other bleeding sources; although no confirmed PE events were documented, asymptomatic PE may have been underdetected. Fifth, the cohort was restricted to severe HICH patients undergoing surgery, limiting generalizability to other populations. Finally, the choice of 48 h as the time threshold was based on clinical and literature-based considerations. In future studies with larger sample sizes, sensitivity analyses using alternative time cutoffs (e.g., 24 h, 48 h, 72 h) or treating timing as a continuous variable could be performed to further refine the optimal timing of pharmacological VTE prophylaxis.

## 6 Conclusion

In this study, early use of LMWH was associated with a significantly reduced risk of lower extremity DVT, and no significant increase in intracranial rebleeding or gastrointestinal bleeding was observed. This supports the consideration of early pharmacological prophylaxis in such patients under close monitoring. However, given the limited sample size and number of events, these safety findings should be interpreted with caution. Future multicenter, prospective randomized controlled trials with larger sample sizes are warranted to confirm the safety of this strategy.

## Data Availability

The datasets used and/or analyzed during the current study are subject to the following restrictions: 1. Privacy and confidentiality restrictions: The datasets contain sensitive personal health information of the patients. Public dissemination is strictly prohibited by China’s Personal Information Protection Law, Regulations on the Management of Human Genetic Resources, and the hospital’s internal data security policies to protect participant confidentiality. 2. Ethical and institutional restrictions: The study protocol and data use agreement were approved by the Ethics Committee of The First Affiliated Hospital of Chengdu Medical College (Approval No. 2025CYFYIRB-BA-162) under the condition that data access is controlled and limited to authorized research purposes only. A waiver of informed consent was granted specifically for retrospective analysis of de-identified records, not for public data sharing. 3. Data sensitivity: Even in de-identified form, the clinical details (e.g., severe neurological injury, precise surgical timelines, and complication outcomes) could potentially be linkable to individuals within a small, specialized patient population, posing a residual re-identification risk. 4. Conditional access pathway: To balance research transparency with the above obligations, a de-identified analytical dataset can be made available to qualified researchers for purposes of verification or secondary analysis, subject to a formal approval process. Requests must be submitted to the Corresponding author (XX, xun.xia@aliyun.com), and will require: A scientifically justified research proposal. Approval from the aforementioned Ethics Committee. A signed data use or material transfer agreement stipulating compliance with confidentiality, non-redistribution, and destruction after use. Requests to access these datasets should be directed to XX, xiaxuns@126.com.

## Author’s note

All the authors take responsibility for all aspects of the reliability and freedom from bias of the data presented and their discussed interpretation.
